# Interformat equivalence between paper and electronic administrations of the self-assessment anhedonia scale in a substance use disorder clinical sample

**DOI:** 10.3389/fpsyg.2026.1753551

**Published:** 2026-05-01

**Authors:** Yolibel Sanjuán, Valentín Estévez-Pérez, Gerardo Flórez, Mercedes Martínez-Montero, Indalecio Machado-Carrera, Adolfo Piñón-Blanco, Luis Iglesias-Rejas, Tania Rivera-Baltanás, María Sánchez-Luaces, Jose Campos-Pérez, Marta López-García, Jose Manuel Olivares, Carlos Spuch

**Affiliations:** 1 Translational Neuroscience Research Group, Galicia Sur Health Research Institute (IIS-Galicia Sur), SERGAS-UVIGO, Vigo, Spain; 2 Addictive Behaviour Unit, Psychiatry Service, Ourense University Hospital Complex (CHUO), Galician Health Service (SERGAS), Ourense, Galicia, Spain; 3 Centre for Biomedical Research in the Mental Health Network (CIBERSAM), Madrid, Spain; 4 Research Department, Citizens' Association for the Fight Against Drugs (ACLAD), A Coruña, Spain; 5 Red de Investigación en Atención Primaria de Adicciones (RIAPAD), ISCIII, Barcelona, Spain

**Keywords:** anhedonia, electronic health records, mental health assessment, psychometrics, reproducibility of results, substance-related disorders, surveys and questionnaires, telemedicine

## Abstract

**Background:**

The increasing integration of digital tools in clinical practice and research has led to the widespread use of electronically administered questionnaires. However, when a paper-and-pencil (paper-based) instrument is digitized, its original psychometric properties may not be preserved; assessing interformat reliability is essential to ensure data validity.

**Objective:**

To evaluate the interformat reliability and psychometric equivalence between the original paper-and-pencil Self-Assessment Anhedonia Scale (SAAS-p) and its electronic version (SAAS-e) in individuals with substance use disorders (SUDs).

**Methods:**

A fixed-order crossover design was used. Fifty-five adults diagnosed with SUDs were recruited from two Addiction Behavior Units during psychotherapeutic treatment. All participants completed both the SAAS-p and SAAS-e. Internal consistency (Cronbach’s *α* and McDonald’s *ω*), interformat reliability (intraclass correlation coefficients, ICCs), and mean score differences were analyzed.

**Results:**

Both SAAS formats demonstrated excellent internal consistency across the Global Scale, subscales, and dimensions (*α*/*ω* > 0.80, mostly > 0.90). Interformat reliability was high, with all ICCs exceeding 0.97. No significant differences in mean scores were detected between formats except for the Intensity subscale, which showed slightly higher scores in the electronic version; this difference remained within acceptable equivalence thresholds and lacked clinical significance.

**Conclusion:**

The SAAS-e and SAAS-p show strong psychometric equivalence in patients with SUDs, supporting the reliability of the electronic version and extending evidence of the scale’s validity to a heterogeneous clinical population.

## Introduction

The use of technology to support clinical practice and research has grown substantially in recent decades, fostering the development of e-health and digital mental health solutions. This trend has been accompanied by the increasing use of digitally administered questionnaires as a means of gathering psychological information (Austin et al., 2006).

Computer-based and Internet testing (CBIT) and Internet-administered testing offer several advantages over traditional paper-and-pencil formats. From a data management perspective, they reduce ambiguity, enhance data integrity, and minimize missing values through automated collection procedures (Andersson et al., 2008; Gwaltney et al., 2008; Hedman et al., 2010). Additionally, digital administration facilitates data access, storage, and transfer, while enabling automated scoring and reducing human error (Holländare et al., 2010). These formats also simplify administration, reduce costs and environmental burden, and decrease investigator workload. Furthermore, digital questionnaires may reduce social desirability bias and embarrassment when addressing sensitive topics, although this effect may depend on contextual factors (Erdman et al., 1992).

Although transferring a paper-based questionnaire to a digital format may appear straightforward, equivalence between formats cannot be assumed. While several studies have reported comparable scores between paper-based and computerized versions (Andersson et al., 2008; Gwaltney et al., 2008; Buchanan, 2003), differences in administration format may introduce response variation and compromise previously established psychometric properties. Such variation may arise from interface characteristics, item presentation, layout, or respondents’ perceptions of anonymity and data security (Alfonsson et al., 2014). In addition, technology-related factors such as computer anxiety (Schulenberg and Yutrzenka, 2002), computer aversion (Tseng et al., 1998), and varying levels of digital literacy may influence responses, particularly in measures of negative affect. Response variation across formats may have important clinical implications, especially when questionnaires are used for screening or clinical decision-making. The International Test Commission guidelines recommend evaluating measurement equivalence when adapting psychological instruments to new administration formats to ensure reliability and validity of the collected data (Ribot, 1896).

Anhedonia, originally described by Théodule Ribot as the inability to experience pleasure, is a transdiagnostic construct observed across multiple psychiatric conditions, including depression, schizophrenia, and SUD (Destoop et al., 2020). In SUD, anhedonia has been associated with early substance use initiation, increased severity of addiction, and the transition from recreational to compulsive drug use (Hatzigiakoumis et al., 2011; Heitzeg et al., 2014). Moreover, anhedonia has been shown to predict craving intensity, relapse risk, and poorer treatment outcomes in individuals with SUD (Pizzagalli, 2020; Stull et al., 2022; Olivares et al., 2005). High levels of anhedonia may also contribute to reduced engagement in treatment and diminished responsiveness to psychosocial interventions. Therefore, accurate assessment of anhedonia in clinically heterogeneous SUD samples, including individuals with comorbid psychiatric disorders, is particularly relevant for optimizing treatment planning and monitoring outcomes.

Recent research has increasingly conceptualized anhedonia as a core mechanism underlying addiction maintenance and recovery outcomes in substance use disorders. Neurobehavioral models propose that chronic substance exposure disrupts reward processing systems, reducing sensitivity to natural reinforcers and increasing reliance on drug-related rewards. Empirical studies support this framework, showing that higher levels of anhedonia are associated with greater addiction severity, polysubstance use, and psychiatric comorbidity in individuals with SUD (Stull et al., 2022). Moreover, longitudinal research has demonstrated that elevated anhedonia during early treatment is associated with increased treatment attrition and poorer engagement, suggesting that hedonic dysfunction may interfere with recovery processes (Rabinowitz et al., 2023). Consistent with these findings, systematic reviews have reported that anhedonia predicts stronger craving, higher relapse risk, and reduced likelihood of sustained abstinence across multiple substance types (Garfield et al., 2014). More recent evidence further indicates that substance-specific patterns of anhedonia, such as those observed in cannabis use disorder, are linked to altered reward sensitivity and functional impairment, reinforcing the clinical relevance of assessing hedonic functioning in addiction populations (Poyatos-Pedrosa et al., 2024). Together, these findings highlight anhedonia as a clinically meaningful target and prognostic marker in SUD, underscoring the importance of reliable and scalable tools for its assessment, such as the electronic version of the Self-Assessment Anhedonia Scale (SAAS).

The SAAS is a visual analogue scale (VAS) designed to measure anhedonia (Chapman et al., 1976). Originally developed in a paper-based format, the SAAS consists of 27 self-report items assessing physical, intellectual, and social anhedonia across three dimensions: intensity, frequency, and change over time. This latter dimension represents a distinctive feature compared with widely used scales such as the Snaith-Hamilton Pleasure Scale (Gard et al., 2006), the Chapman Revised Physical Anhedonia Scale and Chapman Revised Social Anhedonia Scale (Rizvi et al., 2015), the Temporal Experience of Pleasure Scale (The International Test Commission, 2005), and the Dimensional Anhedonia Rating Scale (Nunnally and Bernstein, 1994), none of which explicitly capture perceived changes in anhedonia over time.

The SAAS has demonstrated robust psychometric properties in previous validation studies. The original scale development reported high internal consistency, with Cronbach’s alpha coefficients ranging between 0.90 and 0.92 across subscales, indicating strong internal reliability. The SAAS was designed as a multidimensional instrument assessing physical, intellectual, and social anhedonia across three dimensions: intensity, frequency, and change, supporting its construct validity and clinical interpretability. In addition, the inclusion of the “change” dimension provides a distinctive feature that allows assessment of perceived variations in hedonic functioning over time, enhancing sensitivity to clinical change. Previous studies have also reported good convergent validity with other measures of anhedonia and mood-related constructs, as well as sensitivity to clinical differences across psychiatric populations. These findings support the SAAS as a reliable and clinically meaningful instrument for assessing anhedonia, particularly in populations where changes in reward processing and motivational functioning are central features, such as individuals with substance use disorders (Olivares et al., 2005).

The SAAS has demonstrated good internal consistency, factorial validity, and sensitivity to clinical change in previous studies (Chapman et al., 1976). Moreover, its use in populations with SUD has shown its ability to detect clinically meaningful variations in hedonic functioning and to capture dimensions relevant to addiction severity and treatment response. However, the manual scoring procedure of the original paper-based version may limit its practical use in clinical and research settings. To address this limitation, an electronic version of the SAAS with automated scoring was developed.

### Objectives

The present study aims to examine the psychometric equivalence between the paper-based and electronic versions of the SAAS in a clinically heterogeneous SUD sample, including individuals with and without comorbid psychiatric disorders. Specifically, we aimed to: (1) assess interformat reliability between paper-based and electronic administrations; (2) compare internal consistency across formats; (3) evaluate agreement between formats at the subscale and total score levels; and (4) explore convergent validity of the electronic version through its associations with relevant clinical variables. We hypothesized that the electronic version would demonstrate comparable reliability and psychometric properties to the original paper-based format in this clinical population.

## Methods

### Study design and recruitment

The sample consisted of 55 participants undergoing treatment for SUD at two outpatient facilities within the Ourense University Hospital Complex (Ourense, Spain). Participants were recruited by clinicians from outpatient addiction treatment programs. Assessments were conducted during the early phase of treatment, specifically within the first 2 weeks following treatment initiation, to minimize variability associated with treatment-related clinical changes.

Inclusion criteria required a diagnosis of SUD according to the International Classification of Diseases, 10th Revision (ICD-10), with or without comorbid psychiatric disorders. Exclusion criteria included illiteracy, intellectual disability, or any condition that could compromise comprehension of instructions or the feasibility of completing the assessment protocol.

The study was approved by the Ethics Committee of Pontevedra-Vigo-Ourense (reference number 2023/212). All participants provided written informed consent after receiving a full explanation of the study procedures.

### Sample size determination

Sample size was determined based on recommendations for reliability studies using intraclass correlation coefficients (ICC). Previous methodological literature suggests that a minimum sample of 30–50 participants is generally sufficient to estimate reliability coefficients with adequate precision, particularly when high ICC values (>0.80) are expected (Walter et al., 1998). Given that the present study aimed to assess interformat equivalence using ICC analyses and that prior research on interformat reliability of self-report measures has reported high agreement values, a target sample size of approximately 50 participants was considered adequate.

The final sample included 55 participants, which exceeds commonly recommended thresholds for reliability and equivalence studies and allows stable estimation of ICC values, internal consistency coefficients, and paired comparisons between formats. This sample size is also consistent with previous interformat equivalence studies in clinical populations, which typically range between 30 and 60 participants. Therefore, the sample was considered sufficient to provide reliable estimates of interformat agreement between the SAAS-p and SAAS-e.

### Description of participants

The sample consisted of 55 participants, 78.2% men (*n* = 43) and 21.8% women (*n* = 12), with a mean age of 41.2 ± 10.8 years (range: 18–64). The average age of first substance use was 18 ± 6.68 years. All participants had a diagnosis of SUD. The primary substance of use was cocaine in 50.9% of cases (*n* = 28), followed by cannabis in 23.6% (*n* = 13), heroin in 12.7% (*n* = 7), alcohol in 7.3% (*n* = 4), opioid analgesics in 3.6% (*n* = 2), and tobacco in 1.8% (*n* = 1). Regarding the number of substances used, 43.6% (*n* = 24) reported using one substance, 29.1% (*n* = 16) reported using two substances, and 27.9% (*n* = 15) reported using three or more substances. Additionally, 87% (*n* = 48) of the participants reported regular tobacco use. Of the total sample, at least 66% (*n* = 33) had a mental health comorbidity. In terms of education, 54.5% (*n* = 30) had basic level education, 25.5% (*n* = 14) had medium level education, and 20% (*n* = 11) had higher education.

### Procedure

Assessments were conducted individually by a trained psychologist in a quiet office equipped with a computer. After explaining the study protocol, participants completed a brief clinical and sociodemographic interview. They then received standardized instructions on how to complete the SAAS, with particular emphasis on understanding polarity shifts in semantic visual analogue scales. This step is particularly relevant when assessing clinical populations in which attentional, cognitive, and motivational difficulties may influence response accuracy, including individuals with substance use disorders and comorbid psychiatric conditions (McDonald, 1999).

Participants completed both the paper-and-pencil and electronic versions of the SAAS during the same session. A fixed-order administration was used, with the paper-based version completed first, followed by the electronic version after a short interval of approximately 3–5 min. This approach was selected to minimize potential technological barriers and reduce anxiety related to digital interfaces, which could otherwise introduce additional variance unrelated to the construct being measured. Previous research has shown that computer anxiety and digital unfamiliarity may influence responses in computerized psychological assessments, particularly in clinical populations with varying levels of digital literacy (Alfonsson et al., 2014; Schulenberg and Yutrzenka, 2002).

Although a counterbalanced design is often recommended to control for potential order effects, a fixed-order design was chosen to prioritize participant comfort and reduce potential dropout or response bias associated with unfamiliar digital interfaces. Moreover, the short interval between administrations minimized the likelihood of meaningful clinical change between formats. This approach has been used in previous interformat equivalence studies when the goal is to assess functional equivalence under controlled conditions (Andersson et al., 2008; Buchanan, 2003).

Following completion of both versions, participants proceeded with the remaining assessments included in the broader research protocol.

The electronic version was administered through a secure digital platform developed by the Translational Neuroscience Group at the Galicia Sur Health Research Institute.^1^ Each participant was assigned a unique identifier to ensure confidentiality and data protection.

### Materials

#### Self-Assessment Anhedonia Scale (SAAS)

The SAAS is a 27-item self-report visual analogue scale designed to assess anhedonia across three domains: Physical, Intellectual, and Social. Each item is rated along three independent 10-cm lines assessing Intensity, Frequency, and Change, which constitute the subscales.

In the original paper-based version, respondents mark their position on each line, which is subsequently measured manually to the nearest millimeter and converted to a 0–10 score. The Global Score ranges from 0 to 810, with higher scores indicating greater anhedonia.

Previous studies have reported satisfactory internal consistency, factorial validity, and sensitivity to clinical change for the SAAS (Olivares et al., 2005).

#### Electronic version (SAAS-e)

The SAAS-e is a direct digital adaptation of the original paper-based version. The electronic format maintains equivalent item presentation, response format, and user control, while incorporating automated scoring procedures, in accordance with recommendations from the International Test Commission for computerized test adaptations (Ribot, 1896).

At the time of this study, the instructions presented within the electronic interface were a condensed version of those included in the paper-based format. However, all participants received standardized verbal instructions before completing the scale, ensuring functional equivalence across formats.

**Figure 1:**
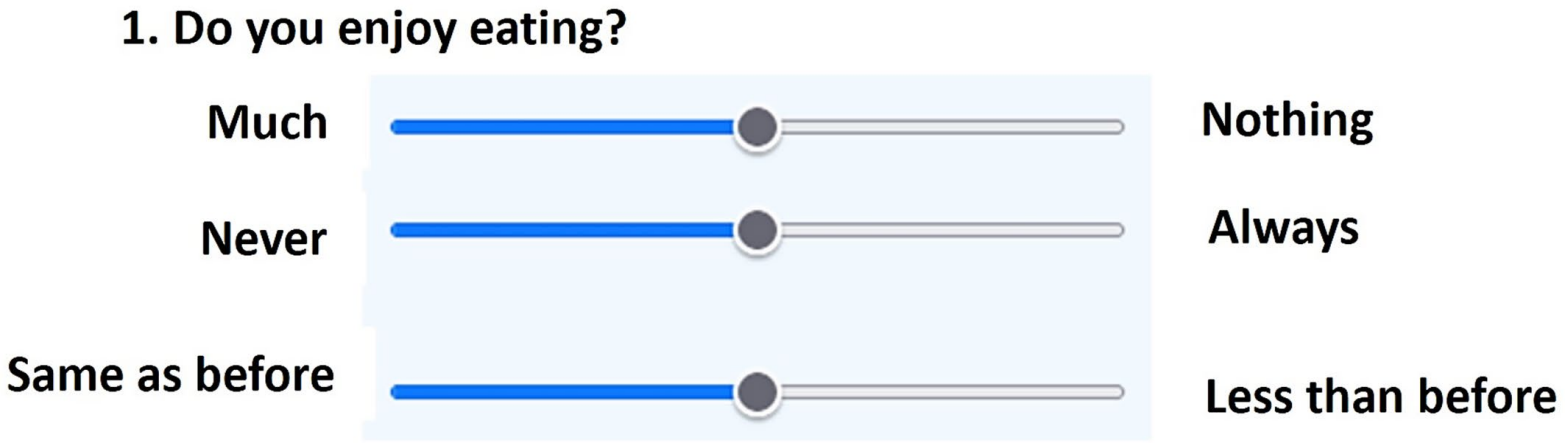
Item format example in the SAAS-e layout.

Following this study, a refined version of the electronic instructions was developed, incorporating minimal wording adjustments designed to reduce cognitive load while maintaining conceptual equivalence. This adaptation was implemented to further enhance clarity in future applications, particularly in clinical populations that may experience attentional or cognitive difficulties. These modifications are consistent with International Test Commission guidelines for digital test adaptation. An example of the electronic layout is shown in Figure 1.

### Statistical analysis

Statistical analyses were performed using Jamovi version 2.6.2.0. In line with recommended guidelines for evaluating computer-based test equivalence, analyses focused on key psychometric properties of both formats, including comparisons of means and standard deviations, estimates of internal consistency, and interformat agreement.

Internal consistency was estimated using Cronbach’s alpha and McDonald’s omega coefficients. These estimates were calculated separately for each format to allow comparison of reliability estimates across versions.

Measures of central tendency and dispersion were calculated to compare mean scores and standard deviations across formats. Intraclass correlation coefficients (ICCs; two-way mixed-effects model, consistency, single measurement) were used to assess interformat reliability. ICCs were selected because they provide a more appropriate measure of agreement between formats than Pearson correlations (Alfonsson et al., 2014).

Wilcoxon signed-rank tests for paired samples were conducted to detect potential systematic differences between formats. This approach was used because high correlations may occur even when systematic differences exist between administrations. Effect sizes were estimated using Cohen’s d.

Additionally, test–retest reliability was estimated by examining agreement between the two administrations conducted within the same session, acknowledging the short time interval between formats. Although this approach does not constitute traditional longitudinal test–retest reliability, it provides an additional indicator of score stability under closely controlled conditions.

A non-parametric approach was adopted when distributional assumptions were not met. A two-sided significance level of 0.05 was used for all analyses.

## Results

The sample consisted of adults undergoing treatment for substance use disorder, predominantly male, with a wide age range and an early onset of substance use. Cocaine was the most commonly reported primary substance, followed by cannabis and other substances, with a substantial proportion of participants reporting polysubstance use and regular tobacco consumption. Most participants presented comorbid mental health disorders and varied educational backgrounds, with basic education being the most frequent level.

### Internal consistency

The internal consistency was assessed for both versions of the scale. The results showed high reliability across all subscales, with Cronbach’s alpha ranging from 0.87 to 0.92 for the SAAS-p and 0.88 to 0.93 for the SAAS-e, and McDonald’s omega ranging from 0.88 to 0.93 for the SAAS-p and 0.89 to 0.93 for the SAAS-e. These results, similar between formats, are also comparable with those reported in previous studies on the development and validation of the original paper-based scale, which yielded Cronbach’s alpha ranging from 0.90 to 0.92. Results can be observed in Table 1.

**Table 1:** Comparison of internal consistency values across SAAS versions—intensity, frequency, and change.

Subscale	SAAS-p *n* = 55		SAAS-e *n* = 55		SAAS-p* *n* = 120
	Cronbach’s a	McDonald’s o	Cronbach’s a	McDonald’s o	Cronbach’s a
Intensity	0.87	0.88	0.88	0.89	0.91
Frequency	0.90	0.91	0.91	0.91	0.92
Change	0.92	0.93	0.92	0.93	0.90

*Scale development study in its original paper-based version; McDonald’s Omega not reported.

In addition to the subscale-level analysis that mirrors the scale development study, we conducted an additional internal consistency assessment for the Global Scale and the Physical, Intellectual, and Social dimensions, all of which showed strong reliability, with Cronbach’s alpha and McDonald’s omega coefficients exceeding 0.80. The Global Scale demonstrated excellent internal consistency across formats (SAAS-p—*α* = 0.954, *ω* = 0.958; SAAS-e—*α* = 0.958, *ω* = 0.961). The Physical dimension yielded (SAAS-p—*α* = 0.920, *ω* = 0.931; SAAS-e—*α* = 0.921, *ω* = 0.932), the Intellectual dimension (SAAS-p—*α* = 0.872, *ω* = 0.877; SAAS-e—*α* = 0.896, ω = 0.900), and the Social dimension (SAAS-p—*α* = 0.826, *ω* = 0.835; SAAS-e—*α* = 0.839, ω = 0.848). A side-by-side summary of these findings is provided in Table 2.

**Table 2:** Comparison of internal consistency values between SAAS versions, Global Scale, and anhedonia dimensions.

Scale/dimensions	SAAS-p *n* = 55		SAAS-e *n* = 55	
	Cronbach’s *α*	McDonald’s ω	Cronbach’s α	McDonald’s ω
Global Scale	0.954	0.958	0.958	0.961
Physical	0.920	0.931	0.921	0.932
Intellectual	0.872	0.877	0.896	0.900
Social	0.826	0.835	0.839	0.848

### Central tendency and dispersion

Mean scores and standard deviations for the SAAS-p and SAAS-e versions were highly similar across all the analyses, with absolute differences in means ranging from 0.3 to 2.0 points and relative differences in variability ranging from 1.0 to 4.9 points. A summary of these values is provided in Table 3.

**Table 3:** Comparison of mean scores and standard deviations between SAAS-p and SAAS-e versions, Global Scale, subscales, and dimensions.

Scale/subscales/dimensions	SAAS-p	SAAS-e	SAAS-p—SAAS-e	SAAS-p—SAAS-e
	M ± SD	M ± SD	Δ Mean	Δ SD
Global Scale	184.2 ± 114.8	185.7 ± 119.7	−1.5	4.9
Intensity	56.9 ± 37.2	58.9 ± 38.8	−2.0	1.6
Frequency	66.9 ± 43.5	66.1 ± 45.7	+0.8	2.2
Change	60.4 ± 50.4	60.7 ± 52.5	−0.3	2.1
Physical	78.6 ± 56.3	79.3 ± 57.4	−0.7	1.1
Intellectual	58.5 ± 38.5	58.9 ± 42.1	−0.4	3.6
Social	47.1 ± 29.0	47.4 ± 30.0	−0.3	1.0

### Intraclass correlation coefficients

The ICC values demonstrated excellent agreement across all the analyses. Specifically, the ICC for the Global Score was 0.993 [95% CI (0.988, 0.996)], while the subscales of Intensity, Frequency, and Change yielded ICCs of 0.992 [95% CI (0.988, 0.996)], 0.985 [95% CI (0.974, 0.991)], and 0.987 [95% CI (0.978, 0.993)], respectively. For the dimensions of Physical, Intellectual, and Social Anhedonia, the ICCs were 0.990 [95% CI (0.984, 0.994)], 0.974 [95% CI (0.955, 0.985)], and 0.987 [95% CI (0.978, 0.993)], respectively. The ICC values for the SAAS versions are summarized in Table 4.

**Table 4:** Intraclass correlation coefficients (ICC) between SAAS versions—Global Scale, subscales, and dimensions.

Scale/subscales/dimensions	*n*	ICC (2,1)	95% confidence interval	
			95% CI lower	95% CI upper
Global Scale	55	0.993	0.988	0.996
Intensity	55	0.992	0.986	0.995
Frequency	55	0.985	0.974	0.991
Change	55	0.987	0.978	0.993
Physical	55	0.990	0.984	0.994
Intellectual	55	0.974	0.955	0.985
Social	55	0.987	0.978	0.993

### Paired sample tests

The Wilcoxon signed-rank tests showed no statistically significant differences between versions for the Global Scale (*W* = 635, *p* = 0.260), Frequency (*W* = 889, *p* = 0.127), and Change (*W* = 421, *p* = 0.087) subscales. Similarly, no significant differences were found for the Physical (*W* = 599, *p* = 0.217), Intellectual (*W* = 678, *p* = 0.896), and Social (*W* = 577, *p* = 0.420) dimensions of anhedonia.

For the Intensity subscale, the Wilcoxon signed-rank test revealed a statistically significant difference between versions (*W* = 371, *p* < 0.001) of moderate effect size (*r* = −0.519), with slightly higher scores observed in the SAAS-e (MD = −1.90). This difference accounts for less than 2 points on a 270-point scale and is accompanied by a very high ICC (0.990). Because an exception was found in the Intensity subscale, which showed a marginally normal distribution (*p* = 0.052), the equivalent parametric test was also conducted for this subscale, yielding consistent results, with a mean difference equal to −2.03, (*t* = −3.05, *p* = 0.004), of a small to moderate effect size (*d* = −0.412). A detailed breakdown of the Wilcoxon test results is provided in Table 5.

**Table 5:** Wilcoxon signed-rank test results between SAAS versions—Global Scale, subscales, and dimensions.

Scale/subscales/dimensions	*n*	Mean difference (MD)	Std. error of difference (SE)	95% CI lower	95% CI upper	Wilcoxon W	*p*	Size effect
Global Scale	55	−1.700	1.851	−4.600	1.050	635	0.260	−0.175
Intensity	55	−1.900	0.665	−3.100	−0.800	371	<0.001	−0.519
Frequency	55	1.650	1.050	−0.500	3.350	889	0.127	0.242
Change	55	−1.500	1.106	−3.200	0.250	421	0.087	−0.285
Physical	55	−0.700	1.060	−2.100	0.600	599	0.217	−0.193
Intellectual	55	0.100	1.249	−1.900	1.550	678	0.896	0.022
Social	55	0.400	1.113	−1.700	0.650	577	0.420	−0.131

## Discussion

Although previous research has explored format equivalence in several psychopathology measures, including anhedonia self-report questionnaires, their theoretical frameworks, item characteristics, and psychometric procedures differ substantially, limiting direct comparability. In the current study, we adhered to the methodological recommendations of Alfonsson et al. (2014), which strengthens the rigor and robustness of our findings.

The results provide strong psychometric support for the reliability and comparability of the SAAS-e. Electronic scores demonstrated comparable reliability indices, similar means and standard deviations, and high interformat correlations with the SAAS-p, in line with international best-practice criteria for interformat equivalence (Holländare et al., 2010; Nunnally and Bernstein, 1994). Importantly, our interpretation of equivalence was based on a combination of complementary indicators, including internal consistency, distributional similarity, intraclass correlation coefficients, and paired comparisons, rather than relying solely on the absence of statistically significant differences between formats.

Internal consistency was high across all SAAS-e subscales and dimensions, with Cronbach’s alpha values exceeding 0.80. These findings satisfy established psychometric standards that define coefficients above 0.80 as very good and above 0.90 as excellent (McDonald, 1999; Koo and Li, 2016). Notably, the reliability of the SAAS-e Intensity, Frequency, and Change subscales closely mirrored SAAS-*p* values and remained consistent with the original validation (*α* = 0.90–0.92). This indicates that the subscales function as coherent measurement units, reinforcing the internal structure of the SAAS-e. These findings also align with those reported by Alfonsson et al. (2014), where most digital instruments surpassed reliability thresholds of 0.70.

Additional analyses indicated excellent reliability for the Global Scale and for the Physical and Intellectual dimensions (*α* and *ω* > 0.90), and strong reliability for the Social dimension (*α* and *ω* > 0.80). Importantly, the high reliability of the Global Scale should not be interpreted as evidence of one-dimensionality. Rather, it reflects a consistent clinical index integrating the inherently multidimensional architecture of anhedonia, a construct now understood to include alterations in anticipatory (“wanting”) pleasure, consummatory (“liking”) pleasure, and motivational and reinforcement-learning components. The total score, therefore, provides a robust global indicator of anhedonia severity while preserving the conceptual richness of the construct.

Regarding the Physical, Intellectual, and Social dimensions, initially defined through expert consensus, the results provide new empirical support for their structural coherence and face validity. Together, these findings strengthen the evidence for the reliability and internal organization of the SAAS-e, particularly in a heterogeneous clinical population.

Comparisons of mean scores and standard deviations revealed minimal differences across formats, with mean differences ranging from 0.3 to 2 points (0.11–0.74% of the total possible range) and variability differences ranging from 1.0 to 4.9 points (0.25–4.3% of scale ranges). These results support the similarity of score distributions between formats. However, it is important to note that the absence of statistically significant differences alone does not constitute formal evidence of equivalence. Instead, these findings should be interpreted alongside additional indicators of agreement, such as high ICC values and minimal absolute differences, which together provide a more comprehensive assessment of interformat comparability.

ICCs exceeded 0.97 for the Global Scale, subscales, and dimensions, indicating excellent interformat reliability (Montoro et al., 2020). In the present study, intraclass correlation coefficients were calculated using a two-way mixed-effects model for consistency. While consistency-based ICCs are commonly used in interformat reliability research, absolute agreement models are often considered more appropriate when evaluating agreement between different modes of administration. Nevertheless, the extremely high ICC values observed suggest strong agreement between formats, and future studies could further strengthen the evaluation by reporting both consistency and absolute agreement models. Similar equivalence has been documented for the Snaith-Hamilton Pleasure Scale (Boateng et al., 2018), further supporting the robustness of digital adaptations in anhedonia assessment.

Wilcoxon analyses confirmed the absence of significant differences between formats for the Global Scale and the Physical, Intellectual, and Social dimensions, as well as for the Frequency and Change subscales. The Intensity subscale showed a small but statistically significant difference, with slightly higher electronic scores (MD = −1.900, *p* < 0.001, *r* = −0.519). However, the absolute difference (2 points; 0.79% of the subscale range) fell well below the ±5% equivalence threshold reported in previous studies (Gwaltney et al., 2008). Interformat agreement remained excellent (ICC = 0.990), and score ranges were highly similar, indicating a negligible and clinically irrelevant shift.

Taken together, the findings support the hypothesis that the SAAS-e is comparable to the SAAS-p and functions as a reliable measure of anhedonia in patients with substance use disorders. Beyond establishing interformat comparability, this study extends the evidence base for the internal consistency of the SAAS in a more heterogeneous clinical population.

In accordance with best-practice recommendations for scale validation (Boateng et al., 2018), methodological decisions balanced psychometric rigor with practical considerations. A fixed-order administration was used to minimize fatigue and avoid confounding effects from difficulties with digital technology, although this design precluded formal assessment of order effects. Additionally, the short interval between administrations helped ensure construct stability but may also have increased the likelihood of recall effects. This trade-off should be considered when interpreting the high agreement observed. A within-format test–retest design was not implemented due to concerns about participant burden within the broader testing protocol.

The study did not include formal measures of response time or usability, as feasibility concerns were limited. However, previous experience suggests that the SAAS-p takes approximately 15 min to complete, while the SAAS-e is typically faster. This finding is consistent with the literature showing that digital assessments can reduce administration errors, streamline data capture, and improve user flow, provided that the digital interface does not alter the cognitive demands of the items. Prior work on ePRO development highlights that interformat equivalence strongly depends on preserving item structure, visual clarity, and the response process, all of which were maintained in the SAAS-e.

Because the study took place under supervised and standardized conditions, caution is warranted when generalizing the findings to unsupervised or ecologically diverse settings. Future research should evaluate the feasibility, usability, and psychometric performance of the SAAS-e as an independent electronic patient-reported outcome, including its use across different environments and levels of digital literacy, and leveraging the newly adapted digital instructions now available.

### Limitations

Several limitations should be considered when interpreting the findings of this study. First, the fixed-order crossover design, in which all participants completed the paper-and-pencil version before the electronic version, may have introduced potential order or carry-over effects. Although this approach was selected to minimize participant burden and avoid confounding related to difficulties with digital technology, respondents may have recalled previous answers when completing the second format, which could have inflated concordance between administrations. In addition, the interval between formats was very short, which helped ensure stability of the construct and minimized the likelihood of true changes in anhedonia. However, this design choice may also have increased the likelihood of recall effects. Therefore, a trade-off exists between maximizing construct stability and minimizing memory-related influences. Future studies should address this limitation by implementing counterbalanced or randomized administration orders and by considering alternative intervals between administrations to better control for potential order and recall effects.

Second, the study did not include within-format test–retest reliability assessments, limiting conclusions about the temporal stability of the SAAS-e. Third, no formal measures of usability or response time were collected. Although prior experience suggested that the electronic format may be faster and more efficient, the absence of direct metrics restricts inferences about user experience and feasibility. Another limitation of the present study is the relatively small sample size, which may limit the generalizability of the findings and the stability of psychometric estimates. Although the sample size is comparable to previous interformat equivalence studies, larger samples would provide more precise estimates of reliability indices, particularly McDonald’s omega, and allow for more robust conclusions regarding the psychometric equivalence between formats. In addition, a larger sample would enable more detailed subgroup analyses, such as comparisons across primary substances, comorbid psychiatric conditions, or demographic characteristics. Future studies with larger and more diverse samples are therefore warranted to confirm and extend the present findings.

Additionally, data were collected under supervised and standardized testing conditions, which may not fully reflect performance in real-world or unsupervised environments. As a result, generalizability to diverse contexts, varying levels of digital literacy, or settings with minimal support remains limited. Furthermore, part of the interpretation of equivalence relied on the absence of statistically significant differences between formats. Although this approach is common in interformat reliability research, non-significant differences alone do not constitute formal evidence of equivalence. While this limitation was mitigated by the inclusion of multiple complementary indicators, including high intraclass correlation coefficients and minimal mean differences, future studies could strengthen conclusions by incorporating formal equivalence testing procedures and evaluating absolute agreement models when assessing agreement between administration modes.

Finally, the sample consisted exclusively of individuals with substance use disorders recruited from two clinical units, which may constrain extrapolation to other clinical populations or community samples. Larger and more diverse samples would allow more precise estimates and broader generalizability.

### Future directions

Future research should extend the current findings by employing counterbalanced or randomized administration orders and exploring different intervals between administrations to better disentangle construct stability from potential recall effects. Such designs would allow a more rigorous evaluation of interformat equivalence and help determine whether the high agreement observed in the present study is maintained under alternative methodological conditions.

Future studies should also incorporate formal equivalence testing approaches, in addition to traditional comparisons of mean differences, to provide stronger statistical evidence of equivalence between formats. Furthermore, reporting intraclass correlation coefficients based on absolute agreement models, alongside consistency-based estimates, would offer a more comprehensive assessment of agreement between administration modes.

In addition, future research should evaluate the performance of the SAAS-e in more ecologically diverse and unsupervised settings, where factors such as digital literacy, device type, and environmental distractions may influence responding. Incorporating formal usability assessments, response-time metrics, and user experience evaluations, ideally comparing both paper and electronic formats, would provide a more comprehensive understanding of feasibility and acceptability, and better inform implementation decisions.

Further studies should also explore the temporal stability of the SAAS-e through within-format test–retest designs and examine measurement invariance across demographic groups, clinical diagnoses, and levels of substance use severity. Expanding validation efforts to other psychiatric populations, particularly those with mood disorders, psychosis, or neurocognitive impairment, would help determine the broader applicability of the scale.

Finally, leveraging the newly adapted digital instructions may facilitate the development of fully independent ePRO systems. Integrating the SAAS-e into mobile platforms or ecological momentary assessment frameworks could offer novel opportunities for monitoring anhedonia dynamically and improving personalized treatment approaches. Ultimately, future work should aim to refine and optimize the digital assessment of anhedonia while ensuring psychometric integrity across contexts and populations.

## Conclusion

The present study provides the first empirical evidence of interformat equivalence between the paper-and-pencil version of the SAAS and its newly developed electronic format (SAAS-e). Across all analyses, including internal consistency, distributional characteristics, interformat agreement, and nonparametric comparisons, the SAAS-e demonstrated excellent psychometric performance, yielding scores that are highly comparable to the traditional format. These findings confirm that the SAAS-e preserves the structural integrity and multidimensional nature of the construct of anhedonia, supporting its use as a reliable clinical and research tool in populations with substance use disorders.

By establishing equivalence between formats, this study advances the growing body of evidence supporting the validity of digital adaptations of psychopathology measures. The results underscore the feasibility of transitioning complex anhedonia assessments to digital platforms without compromising measurement precision, while also aligning with broader trends toward scalable, user-friendly electronic patient-reported outcomes in mental health research.

Nonetheless, the study’s supervised administration and fixed-order design highlight the need for future research to examine the SAAS-e under more ecologically diverse and unsupervised conditions, to evaluate its feasibility, usability, and psychometric stability in independent patient use. Further work should also explore its sensitivity to change and predictive validity across clinical trajectories.

Overall, the SAAS-e emerges as a robust, psychometrically sound, and clinically meaningful digital instrument, offering a reliable alternative to its paper-based counterpart and supporting its integration into contemporary digital health and psychiatric research frameworks.

## Data Availability

The raw data supporting the conclusions of this article will be made available by the authors, without undue reservation.

## Footnotes

1
http://www.bipet.eu.pythonanywhere.com

## References

[ref1] Alfonsson S. Maathz P. Hursti T. (2014). Interformat reliability of digital psychiatric self-report questionnaires: a systematic review. J. Med. Internet Res. 16:e268. doi: 10.2196/jmir.3395, 25472463 PMC4275488

[ref2] Andersson G. Ritterband L. M. Carlbring P. (2008). Primer for the assessment, diagnosis and delivery of internet interventions for (mainly) panic disorder: lessons learned from our research groups. Clin. Psychol. 12, 1–8. doi: 10.1080/13284200802069027

[ref3] Austin D. W. Carlbring P. Richards J. C. Andersson G. (2006). Internet administration of three commonly used questionnaires in panic research: equivalence to paper administration in Australian and Swedish samples of people with panic disorder. Int. J. Test. 6, 25–39. doi: 10.1207/s15327574ijt0601_2

[ref4] Boateng G. O. Neilands T. B. Frongillo E. A. Melgar-Quiñonez H. R. Young S. L. (2018). Best practices for developing and validating scales for health, social, and behavioral research: a primer. Front. Public Health 6:149. doi: 10.3389/fpubh.2018.00149, 29942800 PMC6004510

[ref5] Buchanan T. (2003). Internet-based questionnaire assessment: appropriate use in clinical contexts. Cogn. Behav. Ther. 32, 100–109. doi: 10.1080/16506070310000957, 16291542

[ref6] Chapman L. J. Chapman J. P. Raulin M. L. (1976). Scales for physical and social anhedonia. J. Abnorm. Psychol. 85, 374–382. doi: 10.1037/0021-843X.85.4.374 956504

[ref7] Destoop M. Morrens M. Coppens V. Dom G. (2020). Addiction, anhedonia, and comorbid mood disorder: a narrative review. Front. Psych. 11:556. doi: 10.3389/fpsyt.2020.00556, 31178763 PMC6538808

[ref8] Erdman H. P. Klein M. H. Greist J. H. Skare S. S. Husted J. J. Robins L. N. . (1992). A comparison of two computer-administered versions of the NIMH diagnostic interview schedule. J. Psychiatr. Res. 26, 85–95. doi: 10.1016/0022-3956(92)90019-K, 1560412

[ref9] Gard D. E. Gard M. G. Kring A. M. John O. P. (2006). Anticipatory and consummatory components of the experience of pleasure: a scale development study. J. Res. Pers. 40, 1086–1102. doi: 10.1016/j.jrp.2005.11.001

[ref10] Garfield J. B. Lubman D. I. Yücel M. (2014). Anhedonia in substance use disorders: a systematic review of its nature, course and clinical correlates. Aust. N. Z. J. Psychiatry 48, 36–51. doi: 10.1177/0004867413508455, 24270310

[ref11] Gwaltney C. J. Shields A. L. Shiffman S. (2008). Equivalence of electronic and paper-and-pencil administration of patient-reported outcome measures: a meta-analytic review. Value Health 11, 322–333. doi: 10.1111/j.1524-4733.2007.00231.x, 18380645

[ref12] Hatzigiakoumis D. S. Martinotti G. Di Giannantonio M. Janiri L. (2011). Anhedonia and substance dependence: clinical correlates and treatment options. Drug Alcohol Rev. 30, 590–603. doi: 10.1111/j.1465-3362.2011.00321.x, 21556280 PMC3089992

[ref13] Hedman E. Ljótsson B. Rück C. Furmark T. Carlbring P. Lindefors N. . (2010). Internet administration of self-report measures commonly used in research on social anxiety disorder: a psychometric evaluation. Comput. Hum. Behav. 26, 736–740. doi: 10.1016/j.chb.2010.01.010

[ref14] Heitzeg M. M. Nigg J. T. Hardee J. E. Soules M. Steinberg D. Zubieta J. K. . (2014). Left middle frontal gyrus response to inhibitory errors in children prospectively predicts early problem substance use. Drug Alcohol Depend. 141, 51–57. doi: 10.1016/j.drugalcdep.2014.05.002, 24882366 PMC4106478

[ref15] Holländare F. Andersson G. Engström I. (2010). A comparison of psychometric properties between internet and paper versions of two depression instruments (BDI-II and MADRS-S) administered to clinic patients. J. Med. Internet Res. 12:e49. doi: 10.2196/jmir.1392, 21169165 PMC3057311

[ref16] Koo T. K. Li M. Y. (2016). A guideline of selecting and reporting intraclass correlation coefficients for reliability research. J. Chiropr. Med. 15, 155–163. doi: 10.1016/j.jcm.2016.02.012, 27330520 PMC4913118

[ref17] McDonald R. P. (1999). Test Theory: A Unified Treatment. New Jersey: Lawrence Erlbaum Associates.

[ref18] Montoro C. I. Rodríguez M. R. Reyes del Paso G. A. Duschek S. (2020). Electronic versus paper-and-pencil administration of the Snaith-Hamilton pleasure scale: a comparison of psychometric properties in patients with fibromyalgia. Psychol. Assess. 32, 211–226. doi: 10.1037/pas0000779 31647255

[ref19] Nunnally J. C. Bernstein I. H. (1994). Psychometric Theory. 3rd Edn. New York: McGraw-Hill.

[ref20] Olivares J. M. Berrios G. E. Bousoño M. (2005). Self-assessment anhedonia scale (SAAS). Neurol. Psychiatry Brain Res. 12, 121–133. doi: 10.1037/t00443-000

[ref21] Pizzagalli D. A. (2020). Toward a better understanding of the mechanisms and pathophysiology of anhedonia: are we ready for translation? Biol. Psychiatry 87, 787–796. doi: 10.1016/j.biopsych.2019.07.018, 35775159 PMC9308971

[ref22] Poyatos-Pedrosa C. Bernabe-Valero G. Pelacho-Ríos L. Iborra-Marmolejo I. (2024). Cannabis and anhedonia: a systematic review. Psychiatry Res. 339:116041. doi: 10.1016/j.psychres.2024.116041 38959579

[ref23] Rabinowitz J. A. Ellis J. D. Strickland J. C. Hochheimer M. Zhou Y. Young A. S. . (2023). Patterns of demoralization and anhedonia during early substance use disorder treatment and associations with treatment attrition. J. Affect. Disord. 335, 248–255. doi: 10.1016/j.jad.2023.05.029, 37192690 PMC10330426

[ref24] Ribot T. (1896) La psychologie des Sentiments Felix Alcan Available online at: https://archive.org/details/b21294124/page/n3/mode/2up (Accessed May 21, 2024).

[ref25] Rizvi S. J. Pizzagalli D. A. Sproule B. A. Kennedy S. H. (2015). Assessing anhedonia in depression: potentials and pitfalls. Neurosci. Biobehav. Rev. 46, 579–590. doi: 10.1016/j.neubiorev.2014.10.001 PMC4856554 26959336

[ref26] Schulenberg S. E. Yutrzenka B. (2002). The equivalence of computerized and paper-and-pencil psychological instruments: implications for measures of negative affect. Psychol. Assess. 14, 422–429. doi: 10.1037/1040-3590.14.4.422 10495816

[ref28] Stull S. W. Bertz J. W. Epstein D. H. Bray B. C. Lanza S. T. (2022). Anhedonia and substance use disorders by type, severity, and with mental health disorders. J. Addict. Med. 16, e150–e156. doi: 10.1097/ADM.0000000000000891, 34282082 PMC8761228

[ref29] The International Test Commission (2005). International guidelines on computer-based and internet-delivered testing. Int. J. Test. 6, 143–171. doi: 10.1207/s15327574ijt0602_4

[ref30] Tseng H. M. Tiplady B. Macleod H. A. Wright P. (1998). Computer anxiety: a comparison of pen-based personal digital assistants, conventional computer and paper assessment of mood and performance. Br. J. Psychol. 89, 599–610. doi: 10.1111/j.2044-8295.1998.tb02705.x, 9854805

[ref31] Walter S. D. Eliasziw M. Donner A. (1998). Sample size and optimal designs for reliability studies. Stat. Med. 17, 101–110. doi: 10.1002/(sici)1097-0258(19980115)17:1<101::aid-sim727>3.0.co;2-e 9463853

