# Correction: Interformat equivalence between paper and electronic administrations of the self-assessment anhedonia scale in a substance use disorder clinical sample

**DOI:** 10.3389/fpsyg.2026.1906724

**Published:** 2026-07-10

**Authors:** Yolibel Sanjuán, Valentín Estévez-Pérez, Gerardo Flórez, Mercedez Martínez-Montero, Indalecio Machado-Carrera, Adolfo Piñón-Blanco, Luis Iglesias-Rejas, Tania Rivera-Baltanás, Maria Sánchez-Luaces, Jose Antonio Campos-Pérez, Marta López-García, Jose Manuel Olivares, Carlos Spuch

**Affiliations:** 1 Translational Neuroscience Research Group, Galicia Sur Health Research Institute (IIS-Galicia Sur), SERGAS-UVIGO, Vigo, Spain; 2 Addictive Behaviour Unit, Psychiatry Service, Ourense University Hospital Complex (CHUO), Galician Health Service (SERGAS), Ourense, Galicia, Spain; 3 Centre for Biomedical Research in the Mental Health Network (CIBERSAM), Madrid, Spain; 4 Research Department, Citizens' Association for the Fight Against Drugs (ACLAD), A Coruña, Spain; 5 Red de Investigación en Atención Primaria de Adicciones (RIAPAD), ISCIII, Barcelona, Spain

**Keywords:** anhedonia, electronic health records, mental health assessment, psychometrics, reproducibility of results, substance-related disorders, surveys and questionnaires, telemedicine

The author contributions statement was erroneously given as YS: Writing – original draft, Writing – review & editing. VE-P: Methodology, Investigation, Data curation, Formal analysis, Writing – review & editing. GF: Writing – review & editing, Methodology, Investigation, Formal analysis, Data curation. MM-M: Methodology, Investigation, Writing – review & editing. IM-C: Funding acquisition, Supervision, Methodology, Writing – review & editing, Data curation, Conceptualization, Investigation. AP-B: Conceptualization, Data curation, Formal analysis, Methodology, Software, Writing – review & editing, Investigation, Supervision, Writing – original draft. LI-R: Writing – review & editing, Software, Data curation. TR-B: Formal analysis, Software, Writing – review & editing, Writing – original draft, Data curation, Methodology, Visualization, Investigation, Conceptualization, Validation, Supervision. MS-L: Formal analysis, Data curation, Methodology, Validation, Investigation, Writing – review & editing, Supervision, Conceptualization. JC-P: Supervision, Investigation, Validation, Funding acquisition, Writing – review & editing, Resources, Visualization. ML-G: Visualization, Resources, Validation, Writing – review & editing, Funding acquisition. JO: Visualization, Methodology, Data curation, Investigation, Supervision, Conceptualization, Validation, Project administration, Software, Funding acquisition, Resources, Writing – review & editing, Writing – original draft, Formal analysis. CS: Writing –original draft, Writing – review & editing.

The correct author contributions statement is

